# Editorial: Psychoneuroendocrinology of Psychosis Disorders

**DOI:** 10.3389/fpsyt.2020.607590

**Published:** 2020-11-09

**Authors:** Grazia Rutigliano, Boris Chaumette, Mary V. Seeman

**Affiliations:** ^1^Department of Pathology, University of Pisa, Pisa, Italy; ^2^Université de Paris, Institut de Psychiatrie et Neurosciences de Paris (IPNP), INSERM U1266, GHU Paris Psychiatrie et Neurosciences, Paris, France; ^3^Department of Psychiatry, McGill University, Montréal, QC, Canada; ^4^Department of Psychiatry, University of Toronto, Toronto, ON, Canada

**Keywords:** neuroendocrine system, immune-inflammatory system, hypothalamus, cortisol, insulin resistance, stress, schizophrenia, schizoaffective disorder

The pathophysiology of psychotic disorders is complex and imperfectly understood. Clinical evidence and animal research link psychotic disorders to multiple systems beyond neuropsychology, in particular the neuroendocrinological and immune-inflammatory systems. That a relationship exists between hormones and the brain has been observed in clinical practice for several centuries. Already back in 1891, Emil Kraepelin hypothesized an association between hormones and dementia praecox, the name he gave to the disease now known as schizophrenia (1). A further insight into this association came from Hoskins (2), who analyzed post-mortem tissues and attempted to treat schizophrenia with glandular extracts (2). The discovery in the 1970s of hypothalamic hormones represented a fundamental milestone in this understanding, which reached a peak when the pituitary gland entered the stage in the 1980s. The notion that neurotransmitters regulate pituitary hormone release via hypothalamic hormones is illustrated in the catchphrase, “the pituitary is the window to the brain” (3).

At present, more than a hundred hormones have been identified, all with complex and partially undetermined actions. Pituitary, thyroid, adrenal, and gonadal dysfunctions are all known to result, on occasion, in psychotic symptoms. Hormones can be briefly involved in the pathophysiology of psychotic disorders through short-term activation that temporarily modifies neuronal function [e.g., adrenocorticotropic hormone (ACTH) release after a stressful stimulus], or through early organizing effects that result in long-lasting structural change (e.g., altered formation of hypothalamic nuclei during fetal life).

Hormones interact broadly with key neurotransmitters implicated in the development of psychosis, namely dopamine, serotonin, glutamate and GABA, the downstream effect being striatal dopamine over-reactivity (4), the most well-established pathophysiological mechanism underlying psychosis. Abnormalities in the hypothalamus-pituitary-adrenal axis (HPA) and hypothalamus-pituitary-thyroid axis (HPT), for instance, are consistently found in people suffering from psychotic disorders, including during early phases of the disorder. In this Special Issue, we feature an analysis of the functional status of the HPA and HPT in a naturalistic population of 486 inpatients with schizophrenia and 154 healthy controls in Shanghai (Zhu et al.). This research team found that hormonal levels varied according to diagnosis of schizophrenia, disease stage (e.g., first-episode vs. recurrent) and gender. Also, a weak association was observed between disease severity and cortisol level. Duval et al. used an integrative experimental approach to investigate HPA and HPT reactivity in response to appropriate stimulating/inhibiting drugs in subjects diagnosed with schizophrenia, schizoaffective or bipolar disorder and healthy controls. Although, due to the small sample size, this remains a preliminary finding, the results showed pathophysiological differences between schizophrenia and depression in schizoaffective and bipolar disorder, which were tentatively explained by distinct dopamine and noradrenaline abnormalities.

Hormonal balance is known to be impacted by environmental triggers. According to the neural diathesis-stress model, abnormalities in the HPA result in an exaggerated response to psychosocial stressors, which, in turn, can impact the dopaminergic and glutamatergic systems. This could explain the role of psychosocial stressors as so-called second-hit risk factors in the neurodevelopment trajectories that culminate in psychosis. The issue of the triangular relationship among stress, HPA and psychosis is addressed in three articles of the present Research Topic. Cullen et al. conducted a meta-analysis to study the concordance between naturally-occurring psychosocial stressors and cortisol levels in subjects with psychosis or at ultra-high risk (UHR) for psychosis and in healthy subjects. The authors did not observe any significant differences in cortisol responses to stressors between subjects in the psychosis spectrum and healthy subjects. Their results contradict the classical view that HPA abnormalities in psychosis are caused by increased exposure or increased sensitivity to stress, instead suggesting that cortisol alterations are epiphenomena of global physiological dysregulation. In the same vein, applying a Mendelian randomization model to a longitudinal cohort of 133 UHR subjects, Iftimovici et al. showed that, given the same cortisol levels, conversion to psychosis is more likely in female subjects with relatively high levels of expression of the glucocorticoid receptor NR3C1. It appears, therefore, that hormonal levels are only one piece in a more complex puzzle that involves genetic, epigenetic, and other physiological regulators. In the last two decades, the microbiome has emerged as an important signaling system that modulates the effects of stress exposure on brain development through cross-talk with glucocorticoid hormones and neurotrophins, as beautifully reviewed by Hoffman et al.. One highly stressful experience is admission to the emergency department as a result of an acute mental crisis. Potvin et al. report an increase in peripheral endogenous cannabinoid levels, such as anandamide and oleoylethanolamide, in 107 patients with schizophrenia/schizoaffective disorder experiencing a mental crisis. An increase in cannabinoid level was positively associated with depressive symptoms, possibly mediated by dysregulation of the HPA axis.

Psychotic disorders usually appear shortly after puberty and present differently in male and female subjects. In addition, psychotic symptoms appear to be exacerbated in women during periods of estrogen withdrawal, such as post-partum and para-menopause. Clinical observations such as these have fuelled research into the role of gonadal hormones in the pathogenesis of psychotic disorders. Animal research has shown that gonadal hormones regulate neurodevelopment and plasticity, and they interact with neurotransmitter systems, including the dopaminergic system. Brzezinski-Sinai and Brzezinski have summarized the vast body of knowledge linking gonadal hormone fluctuations and the course of psychotic disorders in women. Their mini-review offers practical suggestions for optimizing psychosis management throughout various stages of female reproductive life. Aside from physiological fluctuations, the gonadal hormone-gonadotropin releasing hormone (GnRH) feedback system can be impaired in pathological conditions, such as the polycystic ovary syndrome (PCOS). PCOS is frequently co-morbid with psychosis and remains a condition that is often recognized late and treated late. Doretto et al. review pathophysiological hypotheses about PCOS and its co-occurrence with psychosis and address the effects of antipsychotic drugs on this condition. The authors emphasize that antipsychotic-induced weight gain, metabolic disturbances and hyperprolactinaemia worsen PCOS symptoms and are much easier to prevent than to treat once they occur. Exogenous hormones interfere with the production of natural hormones so that contraceptives, hormone replacement therapies, and hormonal treatments for infertility may all indirectly impact proneness to psychosis. The systematic review by González-Rodríguez et al. explores the effects on psychopathology of fertility treatments that induce hypoestrogenism. They can exacerbate depressive and psychotic symptoms and act as triggers of mood and psychotic disorders. Despite several anecdotal reports of psychotic symptoms induced or worsened by fertility treatments, proper trials are still lacking. The authors review nine trials, all conducted in non-clinical populations, that focus on mood profile and advocate for future investigations with participants experiencing psychotic disorders.

Patients with schizophrenia-spectrum disorders are known to have a reduced life expectancy, mostly accounted for by cardiovascular comorbidities. It has been proposed that psychotic disorders and cardiometabolic conditions may share genetic liability as well as neuroendocrinological pathogenicity. Investigating the overlap has been difficult because most studies conducted in patients with schizophrenia are confounded by long-term use of antipsychotics as well as by the effects of chronicity—e.g., social isolation, sedentary habits, poor diet, and substance abuse. Studies assessing patients with recent-onset psychosis are in a better position to yield useful information. In this Research Topic, three studies have focused on this topic. Petruzzelli et al. retrospectively analyse clinical records of a precious, albeit small, sample of drug-naïve children and adolescents admitted to their ward with a diagnosis of schizophrenia-spectrum or affective-spectrum disorders. Young patients with a diagnosis of schizophrenia-spectrum disorder had high fasting glucose, fasting insulin, and insulin resistance index HOMA-IR, indicating a potentially higher risk of diabetes mellitus type 2, when compared to patients with affective-spectrum disorders. However, in a sample of 60 patients with recent-onset psychosis and 50 healthy controls, Montalvo et al. did not detect any significant differences in glucose parameters. Nevertheless, they describe an inverse association between glycated hemoglobin (HbA1c)—a marker of long-term exposure to high glucose levels—and several hippocampus- and prefrontal cortex-dependent cognitive domains. Finally, Lis et al. found significant alterations in leptin levels, glucose metabolism, and lipid profile in patients with a first episode of psychosis when compared to healthy controls, indicating an impairment of the adipo-insular axis in early psychosis. Also in this study, the authors observed a negative correlation between a metabolic parameter—leptin in this case—and cognitive performance. No alteration in the adipo-insular axis was detected in unaffected offspring of schizophrenia patients, failing to support the hypothesis of a shared familial liability between psychotic and metabolic disorders. Future studies with larger sample sizes are needed to confirm these findings.

Psychoneuroimmunology is a branch of psychoneuroendocrinology that studies the interrelationships between the immune-inflammatory system and the central nervous system. The role of the immune system in the etiopathogenesis of psychotic disorders is supported by genetic data that identify several genes located in the major histocompatibility complex and involved in antimicrobial defense as susceptibility genes for schizophrenia. Furthermore, there is an epidemiological connection between maternal infections during pregnancy and psychotic disorders. Aguilar-Valles et al. provide a review of the literature that encompasses both clinical evidence and animal models and explores the effect of maternal inflammatory activation on the development of the dopaminergic neurotransmission system.

Our Research Topic highlights the key role of neuroendocrinological and immune-inflammatory factors in the development, maintenance, and outcome of psychotic disorders. The papers summarized here clearly show that body and mind are one, and that a holistic approach is vital for the effective clinical management of patients with psychosis.

## Author Contributions

All authors listed have made a substantial, direct and intellectual contribution to the work, and approved it for publication.

## Conflict of Interest

The authors declare that the research was conducted in the absence of any commercial or financial relationships that could be construed as a potential conflict of interest.
